# IMPT of head and neck cancer: unsupervised machine learning treatment planning strategy for reducing radiation dermatitis

**DOI:** 10.1186/s13014-023-02201-y

**Published:** 2023-01-14

**Authors:** Noufal Manthala Padannayil, Dayananda Shamurailatpam Sharma, Sapna Nangia, Kartikeshwar C. Patro, Utpal Gaikwad, Nagarjuna Burela

**Affiliations:** 1 Department of Medical Physics, Apollo Proton Cancer Centre, 100 Feet Road Tharamani, Chennai, Tamil Nadu 400053 India; 2 Department of Radiation Oncology, Apollo Proton Cancer Centre, 100 Feet Road Tharamani, Chennai, Tamil Nadu India

**Keywords:** Machine learning, AI, K-means, IMPT, Head and neck cancer, Skin toxicity, Proton therapy

## Abstract

Radiation dermatitis is a major concern in intensity modulated proton therapy (IMPT) for head and neck cancer (HNC) despite its demonstrated superiority over contemporary photon radiotherapy. In this study, dose surface histogram data extracted from forty-four patients of HNC treated with IMPT was used to predict the normal tissue complication probability (NTCP) of skin. Grades of NTCP-skin were clustered using the K-means clustering unsupervised machine learning (ML) algorithm. A new skin-sparing IMPT (IMPT-SS) planning strategy was developed with three major changes and prospectively implemented in twenty HNC patients. Across skin surfaces exposed from 10 (S10) to 70 (S70) GyRBE, the skin's NTCP demonstrated the strongest associations with S50 and S40 GyRBE (0.95 and 0.94). The increase in the NTCP of skin per unit GyRBE is 0.568 for skin exposed to 50 GyRBE as compared to 0.418 for 40 GyRBE. Three distinct clusters were formed, with 41% of patients in G1, 32% in G2, and 27% in G3. The average (± SD) generalised equivalent uniform dose for G1, G2, and G3 clusters was 26.54 ± 6.75, 38.73 ± 1.80, and 45.67 ± 2.20 GyRBE. The corresponding NTCP (%) were 4.97 ± 5.12, 48.12 ± 12.72 and 87.28 ± 7.73 respectively. In comparison to IMPT, new IMPT-SS plans significantly (*P* < 0.01) reduced SX GyRBE, gEUD, and associated NTCP-skin while maintaining identical dose volume indices for target and other organs at risk. The mean NTCP-skin value for IMPT-SS was 34% lower than that of IMPT. The dose to skin in patients treated prospectively for HNC was reduced by including gEUD for an acceptable radiation dermatitis determined from the local patient population using an unsupervised MLA in the spot map optimization of a new IMPT planning technique. However, the clinical finding of acute skin toxicity must also be related to the observed reduction in skin dose.

## Introduction

Intensity-modulated proton therapy (IMPT) is increasingly recognised as a superior radiation therapy (RT) treatment for head and neck cancer (HNC) [1, 2]. Dosimetric studies have shown that IMPT reduces doses to numerous organs at risk (OARs) more effectively than modern photon intensity-modulated radiotherapy (IMRT) and volumetric-modulated arc therapy (VMAT) [3, 4]. Proton beam therapy (PBT), particularly IMPT, may reduce the risk of dysphagia, xerostomia, dysgeusia, and hypothyroidism [5–7].

Despite the benefits, a key concern in PBT for HNC is radiation dermatitis (RD), which is principally brought on by a decline in the peak to plateau ratio of the spread-out Bragg peak (SOBP). Patients treated with PBT for Ewing's sarcoma [8], breast [9], and brain and spine [10] have reported experiencing RD to varying degrees, ranging from grade-1 to grade-4. The quality of life (QOL) and treatment results may be significantly impacted by RD, which frequently occurs in the early stages of treatment [11–13].

The main factors that determine the degree of skin toxicity are the radiation dose received by the surface as measured by dose surface histograms (DSH), neoadjuvant and concurrent systemic treatment strategies, and patient and tumour characteristics [14–18]. The DSH/dose volume histogram (DVH) of the skin and its correlation with clinically documented dermatitis in patients treated with photon IMRT/VMAT/Tomotherapy techniques [14–17] and passive scattering proton therapy (PSPT) [18] have been used to construct predictive models for RD. In PBT, DSH/DVH is influenced by the extent of the tumor’s proximity to the skin, proton spot map optimization strategy, proton beam characteristics, and treatment delivery technique [18, 19]. Arjomandy et al.’s experimental surface dose measurement study [20] verified that patients treated with the spot scanning technique (SST) received less exposure to the skin than those treated with PSPT. To the best of our knowledge, there is no study on the association between DSH/DVH and the severity of skin toxicity in HNC patients receiving IMPT. In addition, despite skin toxicity being acknowledged as a clinically important problem, no treatment planning strategy to prevent acute RD has been identified.

In this study, RD was predicted using DSH data collected from a group of HNC patients who had received IMPT. Unsupervised K-means clustering machine learning (ML) algorithm [21] was developed to group patients according to the likelihood that they will develop high, medium, or low grades of RD. In an attempt to reduce RD, a new IMPT optimization technique was developed that incorporates predictive model findings. Finally, the anticipated RD resulting from the new planning strategy was contrasted with the RD of initial patients treated using the old planning strategy.

## Materials and methods

In this study, 64 HNC patients (Men:Female = 52:12; median age, 50 years; range, 21 to 80 years) with various histology who received IMPT were included. Each patient received simultaneous integrated boost (SIB) dosage to their primary tumour and bilateral neck nodes from the IMPT plan. In 19 (29.7%) patients treated for definitive settings, an SIB dose regimen of 70 (primary)/56 (nodes) Gy RBE were delivered over 35 fractions while in the remaining 45 (70.3%) post operative patients 60 (primary)/54 (nodes) Gy RBE were delivered in 30 fractions to the tumour bed. Of the 64 patients, the first 44 patients were used for the NTCP model creations and clustering of grades of NTCP using unsupervised K-means clustering ML algorithm. Whereas, the subsequent 20 patients were prospectively enrolled for a new skin sparing IMPT (IMPT-SS) treatment plan and predicted grades of RD were compared with the original IMPT planning technique. The percentage of patients treated with the two SIB dose regimen were similar in the group of patients enrolled for NTCP modelling and IMPT-SS.

### Original IMPT plan

The RayStation (RaySearch Laboratory, Sweden) treatment planning system (TPS, v9.0), which was clinically commissioned for the ProteusPlus (IBA, Luvalleno) proton therapy system (PTS) outfitted with a dedicated PBS nozzle, was used to optimize the IMPT plans. It can modulate proton energy from 70.2 to 226.2 MeV (range 4.1–32 g/cm^2^) with a corresponding spot size of 6.7 to 3 mm sigma in air and at the isocenter. The range of the proton beam can be reduced further to treat superficial tumour using an add‐on Lexan (density = 1.20 g/cm^2^) range shifter (RS) having a physical dimension of 32 × 40 × 3 cm^3^.

The ProteusPlus proton beam characteristics data used in this study are detailed elsewhere [22, 23]. Each treatment plan was created using four fields: two post obliques (120° ± 10°and 240° ± 10°) with the patient positioning system (PPS) angle inclined at ±30° and two anterior obliques (50° ± 5° and 310° ± 5°) with the PPS angle set at 0°. Laterally placed targets were treated by a pair of ipsilateral oblique (anterior and posterior) beams, whereas centrally placed primary CTVs were treated by all four beams. In every field, a rectangular range shifter (Lexan; density = 1.25 g/cm^2^, physical thickness = 3 cm) mounted on the PBS dedicated nozzle was employed, with its outside surface positioned 3 to 5 cm from the most extended body surface. The clinical target volumes (CTVs) for each plan were robustly optimised to account for set-up errors of ± 3 mm and range uncertainties of ± 3.5%. The RayStation TPS's Monte-Carlo (MC) algorithm was always employed for spot map optimization and volumetric dosage calculation. A statistical dose calculation uncertainty of 1% was utilised with a sampling history of 10,000 ions/spot. A planning physicist, an independent physicist, and the clinical site-specific primary physician performed crucial evaluations of treatment plan on every patient. Cone-beam computed tomography (CBCT) volumetric image guidance was used for the daily verification of patient set-up and monitoring of any internal or external changes in patient anatomy.

### Skin toxicity model

The treatment plan databases of the first 44 patients were retrospectively examined to estimate DSH provided by

1 $$\underset{\mathit{ r}\to 0}{\mathrm{DSH}\left(\mathrm{x}\right)=\mathrm{lim}}\frac{{DVH(x)}_{{skin}_{r}}}{r}$$

Where, ${skin}_{r}$ is the 3 mm cutaneous layer of skin created automatically using an in-house developed code from the body contours of the irradiated area. Body contour was constructed accurately as the external surface of the patient’s CT-images excluding the thermoplastic and other immobilization devices. Irradiated area in this manuscript is the section of body contour receiving at least 10% of the prescription dose. In order to extract the DSH of the 3 mm irradiated cutaneous layers from the treatment plan, a second custom script was developed in the python scripting environment offered at RayStation TPS. An open-source program; Computational Environment for Radiation Therapy (CERR) was used to translate Digital Imaging and Communication in Medicine for Radiotherapy (DICOM RT) plans that also contained DSH into Matlab-readable format (MathWorks, Natick, MA, USA). The percentage of surface (S) receiving doses of X GyRBE (S_x_), such as S10 GyRBE, S20 GyRBE, S30 GyRBE, S40 GyRBE, S50 GyRBE, S60 GyRBE, and S70 GyRBE respectively, was used to access the DSH of the skin in the various grades of RD. Re-defined generalised equivalent uniform dose (gEUD) was calculated for each patient with a known RD grade by

2 $$gEUD[D]={\left(\underset{0}{\overset{\infty }{\int }}rDSH\left(x\right){x}^{1/n}dx\right)}^{n}$$

The normal tissue complication probability (NTCP) of skin was estimated using DSH- based Lyman-Kutcher-Burman (LKB) model represented by

3 $${NTCP}_{LKB}[D]=\sqrt{\frac{1}{2\pi }}{\int }_{-\infty }^{t}{e}^{{-x}^{2}/2}dx$$

where

4 $$t=\frac{gEUD\left[D\right]-{TD}_{50}}{m.{TD}_{50}}$$

The specifics of the new formalism of DSH applied to re-defined generalised equivalent uniform dose (gEUD) for the LKB modelling of skin toxicity were described elsewhere [15]. A Matlab programme was created to determine the NTCP according to G plama et al.’s [15] description of skin toxicity. It was decided, by consensus, to investigate the possibility of including anticipated skin toxicity in the treatment planning process. This was aimed at further utilisation of IMPT capabilities and the development of an optimization strategy for lowering skin toxicity, without lowering the recommended dose to the tumour.

### K-means clustering

In order to avoid the inter-patient variability in assessing the grade of toxicity, an unsupervised ML algorithm, K-means clustering, was used to create the clusters of patients. In K-means, the unlabelled dataset is split up into several groups by clustering. We have used the elbow method to find the number of clusters within our patient data set. The goal of this approach is to cluster the data points in a way that minimises the sum of the squared distances between the data points and the centroid (H) represented by

5 $${\varvec{H}}={\sum }_{h=1}^{K}\sum_{i,i\epsilon {c}_{h}}{\Vert {y}_{i}^{h}-{c}_{h}\Vert }^{2}$$

where ${y}_{i}^{h}$ is the data within the cluster ${c}_{h}$

The 44 patients were clustered based on the NTCP and EUD, which divides the unlabelled dataset into various clustered groups. These clusters were used to find the range of DSH, EUD NTCP of skin toxicities of various grades.

### New skin sparing IMPT (IMPT-SS)

Three significant adjustments were made in order to create a new IMPT treatment planning technique. First, whenever the CTV extend up to the body contour, a new CTV (CTV Eval) was created by subtracting 3 mm from the body contour. Then, while concurrently guaranteeing that CTV 95 ≥ 95% and CTV Eval 98 ≥ 98%, robust optimization criteria for set-up error and range uncertainty were applied to CTV Eval. In addition, the new optimization procedure used the mean EUD and DSH calculated for cluster groups from the initial 44 patients for a clinically tolerable skin toxicity, and the trade-off between CTV coverage and DSH was determined using the pareto function of the multi-criteria-optimization (MCO) method [24]**.** The elimination of the range shifter and setting the PPS angle on the two posterior oblique fields to zero degrees constituted the third adjustment to the new planning method. This method of planning will be referred to as skin sparing IMPT (IMPT-SS) from now on. This strategy was implemented prospectively in the planning of 20 HNC patients.

Dose received by each x% volume (Dx%) of the CTVs and OARs was used to compare the original (IMPT) and new skin sparing (IMPT-SS) treatment plans, and DSH and NTCP were used to assess the skin doses. For CTVs, D98%, D95%, and D1% were evaluated, whereas D1% and Dmean were evaluated for relevent OARs. Wilcoxon signed-rank tests were used to analyse the statistical differences between the two planning methodologies and the relationships between DSH, EUD, and NTCP parameters.

## Results

### Analysis of DSH and clustering

**Fig. 1:**
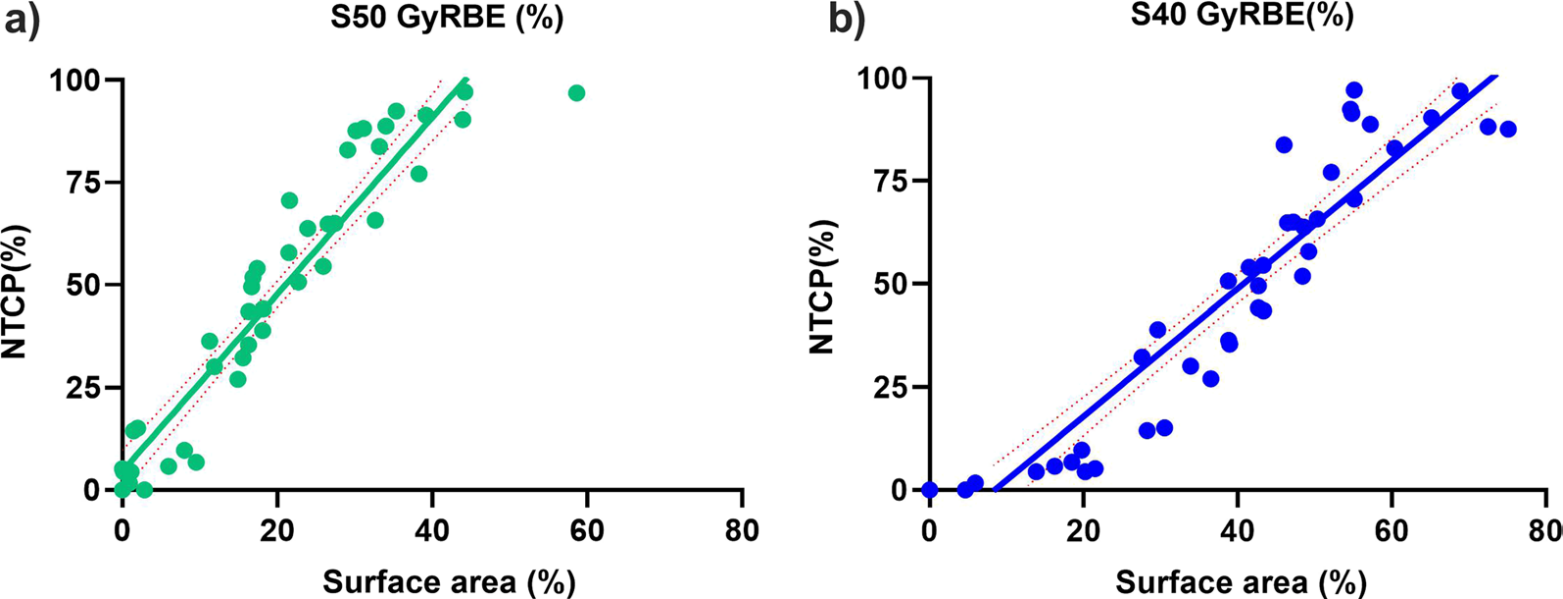
NTCP (%) of skin exposed to different percentages of surface exposed to 50 Gy RBE (**a**) and 40 Gy RBE (**b**)

The calculated NTCP and the skin surface area exposed to various radiation doses (Sx) showed a high association over the whole dose range, based on the correlation heat map. The S50 GyRBE and S40 GyRBE have the strongest correlations (0.95 and 0.94, respectively) with NTCP of skin. As shown in Fig. 1a and b, the NTCP increases nearly linearly with the percentage of skin surface area exposed to 50 Gy RBE and 40 Gy RBE, respectively. As anticipated, the increase in NTCP from 50 Gy RBE was greater than that from 40 Gy RBE for the same surface area of exposure. According to estimates, the rise in skin's NTCP per unit Gy RBE is 0.568 for skin exposed to 50 Gy RBE as opposed to 0.418 for skin exposed to 40 Gy RBE.

**Fig. 2:**
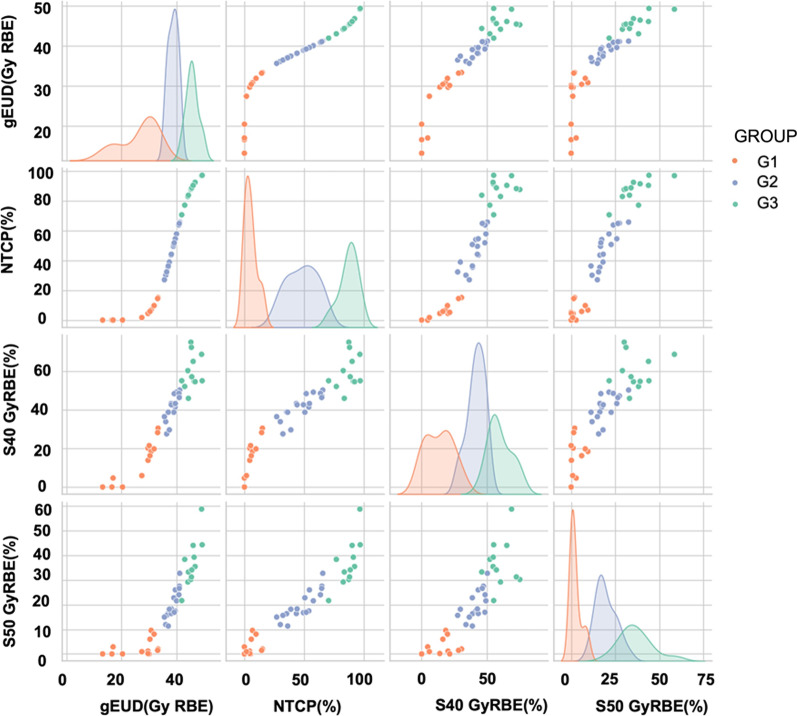
Pair plot analysis of three clusters of patients, GROUP-1 (G1), GROUP-2 (G2) and GROUP-3 (G3). This figure is represented by a grid of axes such that each variable in data will be shared on the y-axis across a single row and on the x-axis across a single column. While along the diagonal histogram showing the univariate distribution of each of the variables

The pair plot representation of the three clusters, group-1 (G1), group-2 (G2), and group-3 (G3), formed by the elbo technique used in the K-means clustering algorithm with gEUD, NTCP (%), S40 GyRBE, and S50 GyRBE, respectively, is shown in Fig. 2. These groups (G1, G2 and G3) are not the same as CTCAE grading system. The findings show a distinct cluster of patients with different NTCPs, with G1 showing a lower NTCP, G2 exhibiting a medium NTCP, and G3 exhibiting a greater NTCP of skin. There were 12 (27%) patients in G3, 14 (32%) patients in G2, and 18 (41%) patients in G1 clusters.

**Fig. 3:**
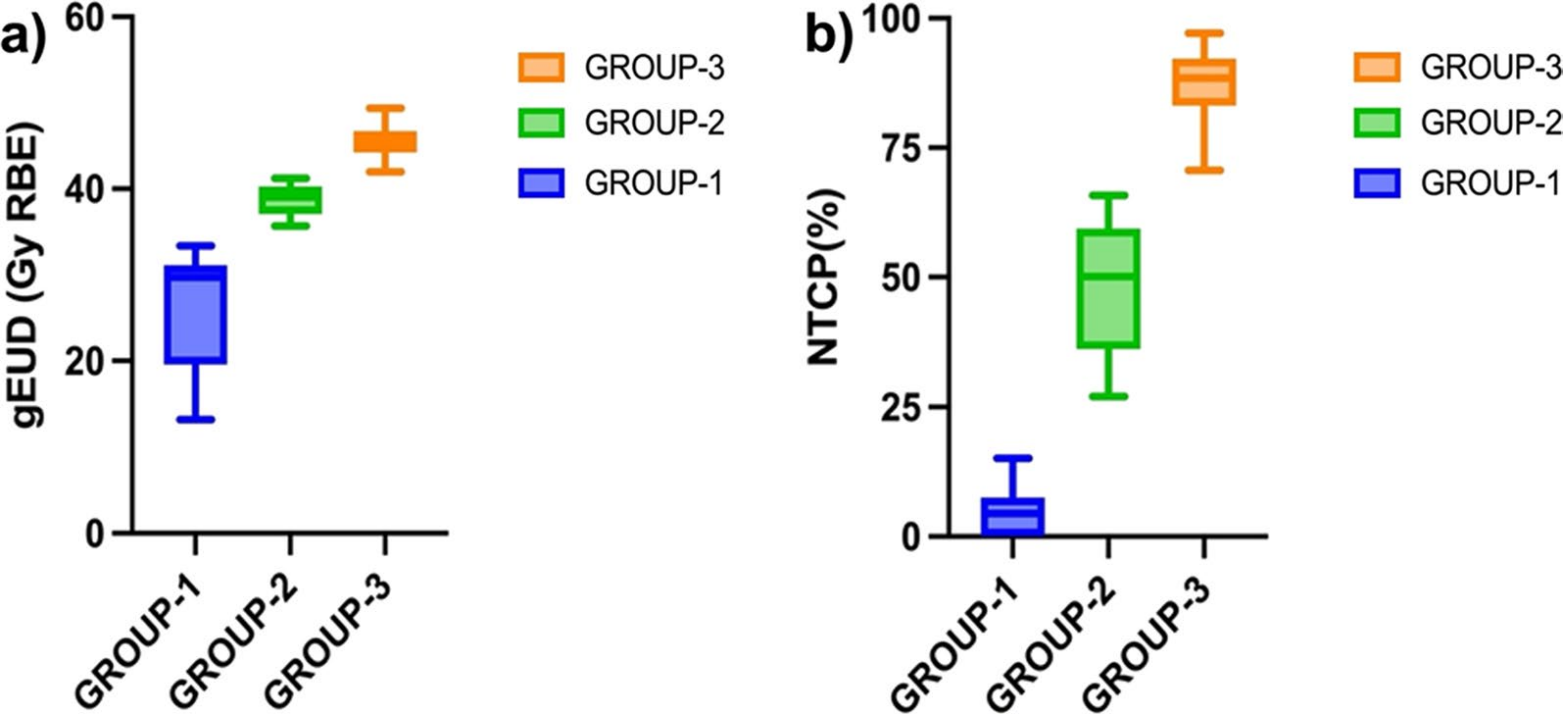
Box plots of **a** generalized equivalent uniform dose (gEUD) and **b** normal tissue complication probability (NTCP) for the three clusters of skin grade of toxicity

Table 1 lists the mean ± SD of the DSH indices of three clusters. For G1, G2, and G3 groups, the percentage of surface area (mean ± SD) receiving 50 Gy RBE was 2.34 ± 3.17, 19.88 ± 5.77, and 36.56 ± 9.43%, respectively. Figure 3 displays the box plot of the EUD (Fig. 3a) and the best fit DSH-based LKB model (Fig. 3b) for the G1, G2, and G3 clusters calculated from the initial 44 patients. The average (± SD) gEUD was 26.54 ± 6.75 Gy RBE for G1, 38.73 ± 1.80 Gy RBE for G2 and 45.67 ± 2.20 Gy RBE for G3 clusters. The corresponding NTCP (%) for G1, G2 and G3 groups were 4.97 ± 5.12, 48.12 ± 12.72 and 87.28 ± 7.73 respectively.

**Table 1:** The overall mean and standard deviation (SD) of the percentage of skin surface receiving X GyRBE (Sx GyRBE) for three clusters (G1, G2 and G3) of patients

Sx Gy RBE(%)	Percentage of surface area receiving Sx Gy RBE for three groups of skin toxicity		
	Group-1 (G1)	Group-2 (G2)	Group-3 (G3)
S10	67.9 ± 22.14	86.03 ± 3.55	93.44 ± 6.72
S20	47.59 ± 19.55	70.32 ± 5.06	86.3 ± 9.00
S30	29.29 ± 16.60	55.78 ± 6.17	75.24 ± 10.0
S40	13.24 ± 10.48	41.55 ± 6.60	59.77 ± 8.83
S50	2.34 ± 3.17	19.68 ± 5.77	36.56 ± 9.43
S60	0.10 ± 0.28	1.74 ± 2.33	8.85 ± 9.63
S70	0.0 ± 0.00	0.02 ± 0.06	0.61 ± 1.35

### Analysis of skin sparing planning technique

**Fig. 4:**
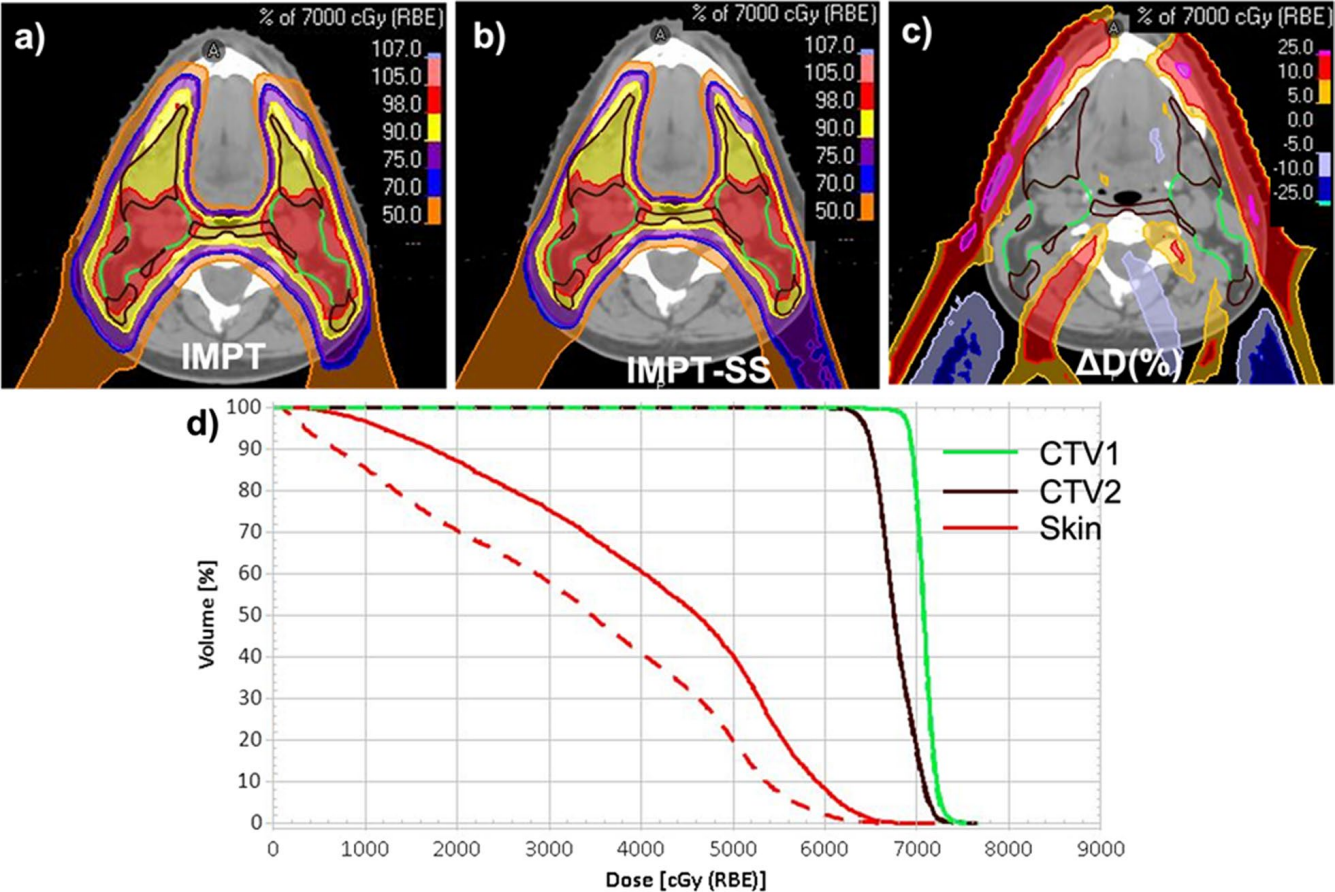
Dose distribution from a representative patient treatment planed using four beams **a** IMPT, **b** IMPT-SS **c** dose difference between IMPT and IMPT-SS in percentage of 70 GyRBE prescribed to primary tumour CTV1 (ΔD(%)) **d** dose volume histogram of CTV1 (green), CTV2 (brown) and skin (read) from the two competing planning approach of IMPT (solid line) and IMPT-SS (dotted line)

The new planning strategy IMPT-SS, when applied prospectively to the 20 patients, met clinical objectives and produced a dose distribution that was nearly identical to that of the original plans, with the exception of skin dose. The comparison of the dose distribution and dosage difference of two planning strategies is shown in Fig. 4. Although both planning procedures cover the same target area, a considerable dosage differential was seen close to the skin. The comparative dosimetric parameters of targets and OARs were displayed in Tables 2 and 3. Table 2 demonstrates that there is no statistically significant difference between the two planning approaches in the target coverage for any of the CTVs. The D98% for CTV eval was above 98% of the dosage in both groups, and the D_95%_ for CTVs was ≥ 95% of the prescribed dose. Similarly, both strategies were successful in achieving comparable OAR sparing, with the exception skin (*P* < 0.001), where the new strategy produced a much lower dose (Table 3).

**Table 2:** The overall mean and standard deviation (SD) dosimetry parameters (D98%, D95%, and D1%) of the CTVs in two treatment planning approaches (IMPT and IMPT-SS), along with the *P* values of the wilcoxon signed rank test

Target	Mean ± SD of D98% from		*P* value	Mean ± SD of D95% from		*P* value	Mean ± SD of D1% from		*P* value
	IMPT	IMPT-SS		IMPT	IMPT-SS		IMPT	IMPT-SS	
CTV1	98.00 ± 1.03	97.86 ± 0.91	0.144	98.85 ± 0.59	98.71 ± 0.72	0.366	105 ± 0.97	105.48 ± 1.54	0.456
CTV2	99.67 ± 0.98	98.73 ± 1.91	0.059	100.53 ± 0.99	100.13 ± 1.64	0.059	105 ± 0.97	105.48 ± 1.54	0.345

*P* < 0.05 is considered statistically significant

**Table 3:** The overall mean and standard deviation (SD) dosimetry parameters (D1%, Dmean) of the OARs in two treatment planning approaches (IMPT and IMPT-SS), along with the *P* values of the wilcoxon signed rank test

Parameters	D1% (Gy RBE)		Dmean (Gy RBE)		*P* value	
	IMPT	IMPT-SS	IMPT	IMPT-SS	D1% (Gy RBE)	Dmean (Gy RBE)
Lips	53.2 ± 18.4	52.5 ± 17.4	24.8 ± 14	21.43 ± 11.99	0.38	0.22
Oral cavity	64.2 ± 5.2	64.4 ± 5.2	39.8 ± 12	39.67 ± 12.14	0.33	0.50
Larynx	61 ± 6.90	61.3 ± 7.0	27.9 ± 10.8	26.9 ± 11.67	0.38	0.35
Mucosa	57.8 ± 19.8	58.3 ± 19.9	32.1 ± 13.8	31.65 ± 13.63	0.31	0.46
Ipsilateral eye	26.3 ± 20.4	26 ± 20.20	7.7 ± 8.4	8.07 ± 8.43	0.49	0.48
Contralateral eye	6 ± 11.50	5.6 ± 10.80	1.7 ± 3.2	1.59 ± 3.38	0.16	0.06
Ipsilateral parotid	59 ± 15.0	59 ± 15.20	42.8 ± 17.8	41.94 ± 17.81	0.34	0.44
Contralateral parotid	39.5 ± 30.3	39.3 ± 30.2	18.3 ± 15.4	17.97 ± 15.11	0.45	0.36
Spinal cord	12.7 ± 6.5	12.2 ± 6.2	3.3 ± 2.3	3.51 ± 2.81	0.46	0.47
Brainstem	24.8 ± 16.1	24.2 ± 16.7	7.7 ± 7.5	7.86 ± 7.52	0.48	0.46
Ipsilateral optic nerve	21.1 ± 22.2	20.7 ± 21.8	13.7 ± 18.5	13.68 ± 18.2	0.45	0.49
Contralateral optic nerve	10.2 ± 15.5	10.1 ± 15.9	4.8 ± 9.7	4.48 ± 9.5	0.43	0.20
Chiasm	13.1 ± 18.2	12.3 ± 17.8	7.5 ± 11.8	7.01 ± 11.44	0.41	0.41

*P* < 0.05 is considered statistically significant

IMPT-SS significantly (*P* < 0.01) reduced the SX GyRBE, gEUD, and associated NTCP, as indicated in Table 4. The mean NTCP values for IMPT-SS were 34% lower than from IMPT. In the IMPT and IMPT-SS plans, the mean ± SD of NTCP was 76.97 ± 26.83% and 42.73 ± 24.55%, respectively. The Wilcoxon signed-rank test's corresponding p values are shown in Table 4 together with the mean and standard deviation for the radiobiological and DSH indices. Both at higher and lower dosages, the IMPT-SS approach shows significantly less dose to the skin. In the IMPT technique, the median result for S20 GyRBE was 84.0% (range: 49–100%), but in the IMPT-SS approach, it was 68% (range: 22–98.4%). Similarly, the median value for IMPT and IMPT-SS plans was 38% (range: 0–87%) and 17% (range: 0–37%), respectively.

**Table 4:** The overall mean and standard deviation (SD) of percentage of the surface receiving X Gy RBE  (S_X_ GyRBE), equivalent uniform dose (EUD) in Gy RBE and normal tissue complication (NTCP) in percentage in the two techniques, along with the *P* values of the wilcoxon signed rank test

Parameters	Difference in percentage of skin surface exposed to various dose level (Sx GyRBE) from old and new planning techniques and corresponding gEUD and NTCP (%)		
	IMPT	IMPT-SS	*P* value
S10 Gy RBE (%)	92.87 ± 5.8	83.05 ± 12.50	< 0.001
S20 Gy RBE (%)	82.25 ± 12.50	68.23 ± 17.26	< 0.001
S30 Gy RBE (%)	69.97 ± 17.26	54.44 ± 19.72	< 0.001
S40 Gy RBE (%)	56.59 ± 20.01	40.70 ± 17.45	< 0.001
S50 Gy RBE (%)	38.68 ± 18.01	18.22 ± 10.69	< 0.001
S60 Gy RBE (%)	5.18 ± 5.40	1.51 ± 2.2	< 0.001
S70 Gy RBE (%)	0.31 ± 0.65	0.02 ± 0.06	0.043
gEUD (Gy RBE)	44.00 ± 6.95	37.40 ± 6.49	< 0.001
NTCP (%)	76.97 ± 26.83	42.73 ± 24.55	< 0.001

*P* < 0.05 is considered statistically significant

## Discussion

Utilizing dosimetric data gathered from our own patient group who were treated for the HNC using the IMPT approach, we were able to establish a relationship between the dose and exposure to skin surface area for varying NTCP of skin. We chose to use the unsupervised k-means clustering machine learning algorithm to classify three group of skin toxicities: G1, G2 and G3. The groups of skin toxicity referred in this study is not the same as the CTCAE grading system. This approach could potentially avoid the variability in the reporting of clinically reported skin toxicities caused by variations in patient and tumor characteristics, concurrent systemic and neoadjuvant treatment regimens, and physician discrepancies. A significant association was found between the percentage of skin surface area exposed to various doses of radiation and observed skin toxicity. The S50 GyRBE was shown to be the most effective DSH indicator for predicting radiation dermatitis, with a mean surface area of 35.24%, correlating well with G3 and 19.7% with G2 patients. In a comparative examination of HNC patients treated with IMRT/VMAT, Kawamura et al. [17] found that V60 Gy was the most important predictive indicator, with V60Gy > 38 cm^3^ being associated with 43.44% of grade-3 dermatitis. The lack of dose build-up characteristics in protons as compared to photons may be the cause of the lower dose limit for the manifestation of higher NTCP of skin.

Although previous studies using photon RT techniques have examined the DSH/DVH and its relationship to the level of skin toxicity and related NTCP, a comparable study using IMPT has not been done. Despite the complicated process involved, we chose DSH over DVH because, in our opinion, DSH represents a clinically realistic natural tool for understanding radiation-induced skin toxicity. Interested readers may refer publication by Palma G et al. [15] for elaborate discussion on the use of DSH over DVH for skin NTCP modelling.

Skin toxicity in IMPT is influenced by a number of treatment planning parameters. The size of the tumour in the beam direction, the planning approach for optimization, the spot energy of the irradiation fields, and the spot map optimization technique. It greatly depends on how dose gradients between the skin and the target are optimised. The posterior non-coplanar beam arrangement was used in the original IMPT plans to enable positioning of the range shifter (35 × 35 cm^2^) from posterior oblique fields closure to patient’s body surface without colliding with the patient or PPS. However, in the new IMPT-SS plans, the PPS rotation was avoided to simplify the treatment planning and delivery process. This necessitates removal of range shifter from the posterior oblique beams. The underdose to sallower section of tumour caused by the removal of range shifter from posterior oblique beams are compensated from the anterior oblique beams of the same side used with range shifter. Using the typical gEUD determined from our own patients for a clinically acceptable degree of skin toxicity, we investigated the potential of spot map optimization to lower skin dose. CTVeval was developed as part of the new treatment planning methodology so that clinical goals are not overly optimised during the robust optimization.

The dose delivered to the CTV and OARs is less affected by set up and range uncertainties while using this method. However, depending on the extent of the uncertainties taken into consideration, it is anticipated that robust treatment planning will lead to greater doses to nearby normal tissues in order to provide acceptable CTV coverage in all robustly optimised error situations [25, 26]. A higher degree of robustness often leads to higher, and occasionally noticeably larger, OAR dosages, especially for setup robustness in HNC. A differential robustness technique was incorporated in our novel skin sparing planning strategy to prevent overdose clouds around the CTV and skin. The inclusion of the average EUD and prediction power of the NTCP of skin acquired from our own patient cohort was the third significant adjustment made in the new planning technique. Since multiple iteration takes a lot of time, MCO was used to create the ability to trade between potential solutions for skin and CTV coverage. The considerable reduction in skin toxicity grade when compared to initial patients treated with the original planning strategy was highly linked with the dosimetric gain in terms of reduction in gEUD and accompanying NTCP. The co-relation between the findings from this predictive model and clinically recorded skin toxicity data will be presented separately.

## Conclusion

It has been established that the k-means unsupervised machine learning algorithm can be used to cluster predicted degrees of skin toxicity based on dose surface histograms produced from patients who underwent IMPT. A skin sparing IMPT treatment planning strategy has been proposed, incorporating three major changes during the robust optimization of CTV. One of the three modifications that significantly lowers skin toxicity is the inclusion of the gEUD derived from the studied patient group for a clinically acceptable grade of skin toxicity into the proton spot map optimization.

## Data Availability

Yes, will be supply when requested.

## Abbreviations

IMPT: Intensity-modulated proton therapy
RT: Radiation therapy
HNC: Head and neck cancer
VMAT: Volumetric-modulated arc therapy
SOBP: Spread-out brag peak
RD: Radiation dermatitis
DSH: Dose surface histograms
DVH: Dose volume histogram
SST: Spot scanning technique
NTCP: Normal tissue complication probabilities
MCO: Multi-criteria-optimization
CBCT: Cone-beam computed tomography
DICOM RT: Digital Imaging and Communication in Medicine for Radiotherapy
gEUD: generalized Equivalent uniform dose
CTV: Clinical target volumes
MC: Monte-Carlo
D1%: Dose to 1% volume
D95%: Dose to 95% volume
D98%: Dose to 98% volume

## References

[1] Leeman JE, Romesser PB, Zhou Y, McBride S, Riaz N, Sherman E, Cohen MA, Cahlon O, Lee N (2017). Proton therapy for head and neck cancer: expanding the therapeutic window. Lancet Oncol.

[2] Beddok A, Vela A, Calugaru V, Tessonnier T, Kubes J, Dutheil P, Gerard A, Vidal M, Goudjil F, Florescu C, Kammerer E, Benezery K, Herault J, Poortmans P, Bourhis J, Thariat J; GORTEC, the 3 French proton centers. Proton therapy for head and neck squamous cell carcinomas: A review of the physical and clinical challenges. Radiother Oncol. 2020; 147:30–39. 10.1016/j.radonc.2020.03.006 32224315

[3] Steneker M, Lomax A, Schneider U (2006). Intensity modulated photon and proton therapy for the treatment of head and neck tumors. Radiother Oncol.

[4] Kandula S, Zhu X, Garden AS, Gillin M, Rosenthal DI, Ang KK, Mohan R, Amin MV, Garcia JA, Wu R, Sahoo N, Frank SJ (2013). Spot-scanning beam proton therapy vs intensity-modulated radiation therapy for ipsilateral head and neck malignancies: a treatment planning comparison. Med Dosim.

[5] Holliday EB, Frank SJ (2016). Proton therapy for nasopharyngeal carcinoma. Chin Clin Oncol.

[6] Vai A, Molinelli S, Rossi E, Iacovelli NA, Magro G, Cavallo A, Pignoli E, Rancati T, Mirandola A, Russo S, Ingargiola R, Vischioni B, Bonora M, Ronchi S, Ciocca M, Orlandi E (2022). Proton radiation therapy for nasopharyngeal cancer patients: dosimetric and NTCP evaluation supporting clinical decision. Cancers (Basel).

[7] Romesser PB, Cahlon O, Scher E, Zhou Y, Berry SL, Rybkin A, Sine KM, Tang S, Sherman EJ, Wong R, Lee NY (2016). Proton beam radiation therapy results in significantly reduced toxicity compared with intensity-modulated radiation therapy for head and neck tumors that require ipsilateral radiation. Radiother Oncol.

[8] Rombi B, DeLaney TF, MacDonald SM, Huang MS, Ebb DH, Liebsch NJ, Raskin KA, Yeap BY, Marcus KJ, Tarbell NJ, Yock TI (2012). Proton radiotherapy for pediatric Ewing's sarcoma: initial clinical outcomes. Int J Radiat Oncol Biol Phys.

[9] Bush DA, Slater JD, Garberoglio C, Do S, Lum S, Slater JM (2011). Partial breast irradiation delivered with proton beam: results of a phase II trial. Clin Breast Cancer.

[10] Moskvin V, Lasley FD, Ray GL (2014). Acute skin toxicity associated with proton beam therapy in spine and brain patients. J Radiat Oncol.

[11] Duncan W, MacDougall RH, Kerr GR, Downing D (1996). Adverse effect of treatment gaps in the outcome of radiotherapy for laryngeal cancer. Radiother Oncol.

[12] Robertson C, Robertson AG, Hendry JH, Roberts SA, Slevin NJ, Duncan WB, MacDougall RH, Kerr GR, O'Sullivan B, Keane TJ (1998). Similar decreases in local tumor control are calculated for treatment protraction and for interruptions in the radiotherapy of carcinoma of the larynx in four centers. Int J Radiat Oncol Biol Phys.

[13] Fesinmeyer MD, Mehta V, Blough D, Tock L, Ramsey SD (2010). Effect of radiotherapy interruptions on survival in medicare enrollees with local and regional head-and-neck cancer. Int J Radiat Oncol Biol Phys.

[14] Pastore F, Conson M, D'Avino V, Palma G, Liuzzi R, Solla R, Farella A, Salvatore M, Cella L, Pacelli R (2016). Dose-surface analysis for prediction of severe acute radio-induced skin toxicity in breast cancer patients. Acta Oncol.

[15] Palma G, Cella L (2019). A new formalism of Dose Surface Histograms for robust modeling of skin toxicity in radiation therapy. Phys Med.

[16] Mori M, Cattaneo GM, Dell'Oca I, Foti S, Calandrino R, Di Muzio NG, Fiorino C (2019). Skin DVHs predict cutaneous toxicity in Head and Neck Cancer patients treated with Tomotherapy. Phys Med.

[17] Kawamura M, Yoshimura M, Asada H, Nakamura M, Matsuo Y, Mizowaki T (2019). A scoring system predicting acute radiation dermatitis in patients with head and neck cancer treated with intensity-modulated radiotherapy. Radiat Oncol.

[18] Palma G, Monti S, Conson M, Xu T, Hahn S, Durante M, Mohan R, Liao Z, Cella L (2020). NTCP models for severe radiation induced dermatitis after IMRT or proton therapy for thoracic cancer patients. Front Oncol.

[19] Moskvin VP, Estabrook NC, Cheng CW, Das IJ, Johnstone PA (2015). Effect of scanning beam for superficial dose in proton therapy. Technol Cancer Res Treat.

[20] Arjomandy B, Sahoo N, Cox J, Lee A, Gillin M (2009). Comparison of surface doses from spot scanning and passively scattered proton therapy beams. Phys Med Biol.

[21] Zhu A, Hua Z, Shi Y, Tang Y, Miao L (2021). An improved K-means algorithm based on evidence distance. Entropy (Basel).

[22] Shamurailatpam DS, Manikandan A, Ganapathy K, Noufal MP, Patro KC, Rajesh T, Jalali R (2020). Characterization and performance evaluation of the first-proton therapy facility in India. J Med Phys.

[23] Noufal MP, Sharma SD, Patro K, Arjunan M, Krishnan G, Tyagarajan R, Rana S, Chillukuri S, Jalali R (2021). Impact of spot positional errors in robustly optimized intensity-modulated proton therapy plan of craniospinal irradiation. Radiol Phys Technol.

[24] Chen H, Craft DL, Gierga DP (2014). Multicriteria optimization informed VMAT planning. Med Dosim.

[25] van de Water S, van Dam I, Schaart DR, Al-Mamgani A, Heijmen BJ, Hoogeman MS (2016). The price of robustness; impact of worst-case optimization on organ-at-risk dose and complication probability in intensity-modulated proton therapy for oropharyngeal cancer patients. Radiother Oncol.

[26] Noufal MP, Widesott L, Sharma SD, Righetto R, Cianchetti M, Schwarz M (2020). The role of plan robustness evaluation in comparing protons and photons plans—an application on IMPT and IMRT plans in skull base chordomas. J Med Phys.

